# The changing faces of autism: The fluctuating international diagnostic criteria and the resulting inclusion and exclusion—A Norwegian perspective

**DOI:** 10.3389/fpsyt.2022.787893

**Published:** 2022-07-28

**Authors:** Sobh Chahboun, Frode Stenseng, Alexander G. Page

**Affiliations:** ^1^Department of Pedagogy, Queen Maud University College, Trondheim, Norway; ^2^Department of Education and Lifelong Learning, The Norwegian University of Science and Technology, Trondheim, Norway; ^3^Department of Social Work, The Norwegian University of Science and Technology, Trondheim, Norway; ^4^ARENA, Centre for European Studies, University of Oslo, Oslo, Norway

**Keywords:** autism spectrum disorder, diagnosis, development, inclusion, identity

## Abstract

The common understanding of autism spectrum disorders (ASD) has gone through a number of permutations since the first description in 1943. Throughout these shifting understandings, there have been a number of behaviors and diagnostic criteria associated with the condition, many of which are missing in the most recent classifications. The rates of diagnoses of autism have increased greatly. However, there is no reason to think there has been any change in occurrence over the last 70 years, suggesting rather an increase in our knowledge and awareness. Autism has been the subject of several misapprehensions and misrepresentations throughout history. This might be due to heterogeneity of the cases. In addition, the diagnosing of autism spectrum disorders is mainly based on observation and behavioral interpretation, and thus dependent on subjective perceptions of the clinicians themselves. This current scoping review article intends to provide a view on the evolution of the concept of autism and the current stance within Norwegian scholarship, and how it is shaped by international discourses and cultural diversities Such changing concepts especially impacts people with the diagnosis, as it can affect their access to social services, as well as their self-identification as people with ASD.

## Introduction

Special education and special needs are immensely varied fields, dealing with issues such as blindness, cerebral palsy, dyslexia, cystic fibrosis or autism spectrum disorder. The profile variability of the autistic spectrum is especially intriguing to many scholars mainly because of the sharp increase in diagnoses in recent years. Most recently, it has been estimated that, among preschoolers in the Oslo area in Norway, 1 in 384 males and 1 in 1,722 females had Autism/ASD (1). The numbers as recently as 2012 were 1 in 730 and 1 in 5,098, respectively, meaning a near doubling for males, and a near trebling for females in just a handful of years (1). Despite these increased rates of diagnosis, we are still largely in the dark when it comes to understanding autism, and in order to capably address the needs of children with autism spectrum disorder properly, we must maintain focus on the autistic enigma.

Autism as a phenomenon has been understood in a number of different ways since its first description in 1943, both by scholars, professionals, laypeople and the individuals with autism themselves. Seventy years separate Kanner's first description of autism, to our current designation of autism spectrum disorders (ASD), and in that time, rates of diagnoses have increased exponentially as better knowledge has improved our detection. However, no cases with ASD are identical and the profiles are extremely heterogeneous. Adding to this complexity are many habitually occurring comorbid conditions and clinical correlations (2, 3). At the same time, public awareness has grown, both in terms of knowledge and information. This is especially relevant during the first years of kindergarten, since an early diagnosis can be critical to get help and support from different relevant institutions. ASD-individuals are frequently portrayed in the media, and some of those diagnosed have formed groups such as the “aspie” societies. Attempting to describe all the various views on autism and how they intersect, build on, contradict and influence each other would be a massive undertaking far beyond the scope of a single article. However, taking the historical changes in psychological understandings as a starting-point, and then exploring how these have affected such areas as clinical practice, ASD self-image, and prejudice in a single national context, would be more manageable.

This article has several purposes, depending on the needs of the reader. Any scholar studying autism would do well to bear in mind that the term has different meanings depending on time and context, which the current article maps out. This is also an attempt to demonstrate the ways in which these definitions filter outward into the public consciousness, affecting how individuals with ASD are seen and how they understand themselves. Lastly, an understanding of the evolution of the concept over time might help us gain an understanding of what the future might bring.

## What is autism?

Autism spectrum disorder (ASD) refers to a range of neurodevelopmental disorders with some common clinical features, but with very difficult etiologic and diagnostic categorization. Most countries today, including the Scandinavian, use the Diagnostic and Statistical Manual of Mental Disorders (DSM-5) released by the American Psychiatric Association (4). ASD is characterized by an impaired social interaction, problems in communication, restricted interests and stereotyped behavior (4). The variation in profiles and potential comorbid conditions results in a complex perception and understanding of the disorder (5–7).

### The etiology of autism spectrum disorder

The etiology of ASD remains unclear. There are no available diagnostic biomarkers to date (8). One possible cause might be an abnormal structure or function of the brain. Several studies using neuroimaging have shown differences in brain shape and structure in children with ASD compared to typically developing peers, especially in the right hemisphere. This is not proof of causation, however, and there are other theories as to what these findings might signify (9–11).

A greatly supported genetic basis is strongly suggested by several reported studies (12). Some patterns or related disabilities have been observed in family members such as siblings, parents, etc. (13) find that a genetic predisposition might have a greater impact on the actual diagnosis, while the environmental factors played a less prominent role. Controversially, we still have not identified a gene causing ASD, but many researchers have found irregular segments in the ASD individual's genetic code and these irregularities might have been inherited (10).

Genovene and Butler (14) focused on the genetic and metabolic grounds causing ASD. They report up to 40% of cromosomic, DNA or mitochondrial related anomalies although they highlight potentially between a 70 and 90% of heritability. They explain this big difference in percentages by the complexity and heterogeneity of the spectrum. Hundreds of different genetic, epigenetic and environmental factors may interact to give such a variety of beavioural and psyquiatric profiles within ASD.

Another possible explanation is that the comorbid conditions that have been observed in individuals with ASD may be underlying causes, and may have triggered or intensified the ASD condition. Alternatively, an instability in the genes affecting the brain's normal development may be causing an atypical structure or functioning of the right hemisphere. There is also a body of work examining possible *in vitro* causes, such as viral infections or exposure to some chemical products such as mercury and a possible inability to metabolize some toxic substances (10, 14).

## The concept and evolution of autism spectrum disorders

The current scoping review starts with a conceptualization of ASD. For the sake of practicality, in this article, we first focus on the history of ASD and the concept's evolution in time, tradition and geography. We also take into consideration how the different diagnostic criteria have been shifting. A large part of the discussion section will focus on language, as this is a key skill often impaired in neurodevelopmental disorders such as ASD. Furthermore, early signs of ASD will be highlighted as we are talking about early childhood education (ECEC) and therefore being aware of these signs will allow to access a potential early intervention program. The Diagnostic and Statistical Manual of Mental Disorders (DSM) and the International Classification of diseases (ICD) are also taken into account as these make up the principal criteria used in Norway, Scandinavia and the rest of the world. However, we focus on the Scandinavian perspectives, Norway in particular, when discussing the contexts of practice, daily life, prejudice and inclusion.

### Scoping on the etiology of ASD

A recent review of the etiology of ASD used changes over time to explore the possible causes for the disorder (13). The authors found that increased knowledge of ASD could only account for part of the increase in diagnoses, and that there had been an actual increase in the prevalence of autistic traits. What causes such traits remains unclear, however, and the ways in which the etiology of ASD has been conceptualized have been influenced by cultural and social changes. These have been grounded on people's preconceptions depending on timeframes and the evolution of different paradigms. However, more and more researcher highlight the concepts of pleiotropy and equifinality when talking about ASD (15–18). This indeed would explain to a certain extent quite well the variability in profiles of the individuals with the spectrum, if we consider that different interplays between genotypes and phenotypes result in such diagnosis.

Originally, there was confusion as to how individuals displaying characteristics of ASD should be classified. The term autism was first used in 1,911 by Eugen Bleuler (19) to refer to a thought disorder that appears in some schizophrenic patients. This conception was extreme in comparison to how the disorder is perceived today, especially in the developed occidental countries. Since the early 40' s, Dr. Leo Kanner, made the first descriptions of the autistic disorder, describing systematically the difficulty of differential diagnosis with other children with disorders coursing similar clinical characteristics (20).

For Kanner (20), individuals with autism were psychiatric patients needed a long-term institutionalization, although he eventually realized that this would be counterproductive to their prospects of improvement. Prior to the formulation of a diagnosis or any possible interventions, Kanner (21), raised the need for a report on the family and social environment in which the child lived, although, such reports would be considered inadequate by today's standards.

Since the first parental movement in the 60' s, autism pushed the boundaries further and allowed the persons in question to participate to a greater degree in the societies and cultures to which they belonged.Gradually, social awareness increased among teachers, doctors and the general public. This increased understanding had an impact on society, leading to the inclusion of these populations in the educational system.

***Talented and functioning individuals within the spectrum***

Almost simultaneously with Kanner (20, 22) identified a group of four children with similar characteristics, although this study remained virtually unknown until it had been translated into English. However, the characteristics of Asperger Syndrom (AS) differed from Kanner's description of autism. For instance, language was not delayed as in the ASD group, the comorbidity with other deficits were more common, and the onset of the syndrome appears relatively late compared to ASD, which was clear already in early infancy. Besides, Asperger (22) observed that the individuals with AS were clumsier, and that the motor skills in these individuals were clearly less developed. Moreover, a possible genetic prevalence was also observed (23). In 1981, Lorna Wing made a review of several reported cases where the common features of AS included the following characteristics (24):

- Absence of empathy
- Impaired social interaction
- Pedantic and monotonic speech
- Impaired non-verbal skills
- Obsession by certain topics
- Poor motor skills

Originally, Asperger syndrome was a “condition” which was only described in male patients, although female patients were identified later. In terms of diagnosed cases, males indeed seem to be more affected, being four times more prevalent than females. However, this may not indicate a real gender imbalance in who actually has the disorder. Rather, it is probable that females, due to better social and communicative skills, may be diagnosed later in life, or even remain undiagnosed (25). The unfortunate result of this is that, while ASD appears to affect the genders equally (26, 27), girls are more likely to go without the appropriate support in their early years.

## Early signs of ASD

Most authors agree that the signifiers for ASD may be detected between the ages of 12 and 18 months (28). This might be more difficult, and for individuals in the higher end of the spectrum, since the incorporation into a school environment may be a milestone, as this makes the difficulties of communication and social interaction become more salient (29, 30).

### A review of the different classifications of autism spectrum disorder

Through time, the concept of ASD has evolved to “fit” daily social demands and reality in general. It is true that each child is a whole world and no child with autism is exactly the same. However, already in the DSM-III (31) some common traits have been established as followed:

- Onset before 30 months of age
- Pervasive lack of responsiveness to other people
- Gross deficits in language development
- If speech is present, peculiar patterns such as echolalia, difficulties in metaphorical language, and pronominal reversal
- Bizarre responses to various aspects of the environment, e.g., resistance to change, peculiar interest in or attachments to animate or inanimate objects
- Absence of delusions, hallucinations, loosening of associations, and incoherence as in Schizophrenia.

The American Psychiatric Association (31) recognized the existence of cases that resemble autism, but which do not meet the diagnostic criteria for this disorder. Existing concerns to address these cases were present in the revision of DSM-III held in 1987. In the DSM-III-R, the “pervasive developmental disorders” included those disorders in which there was a qualitative deterioration in the development of reciprocal social interaction, communication (verbal and non-verbal) and imaginative activity. The most important point made with the DSM diagnostic classification was mainly to distinguish autism from schizophrenia described earlier in the 40' s by Kanner. Therefore, it was possible to reach a consensus on the conception of autism as a behavioral syndrome that affects a wide range of areas, both cognitive, development and emotional, and considering it as a pervasive developmental disorder, as stated in the different nosological classifications.

In 2002, the concept of autism began to take the shape that we know today in terms of definition, understanding and eventual diagnosis. This was the case because several professionals in different areas of specialization have started to consider an interdisciplinary work (32). However, as expected, the outcome from this was that several hypotheses raised from the increase rates of the diagnosis of autism.

In the Scandinavian countries, the percentages of individuals with autism is in a continuous increase, this could perhaps be explained by the increase of awareness or indeed by the impact of the interdisciplinary teams used to diagnose (33). Through the past decade, more and more researchers and clinicians have been investigating the symptomatology of autism. These have tried to establish the clearest possible criteria that would allow for an accurate diagnosis. These include describing the scope and neurobiological potential causes of the disorder, the progressive refinement of psychological explanations and experimental fact, the interrelationship between the above factors and therefore the consideration of autism as a developmental disorder, introducing the concept of autism spectrum, etc. (25). This allows for more nuanced presentations, taking into account the voices of the individuals themselves, as well as their families and organizations both national and international.

After the DSM-III, a new updated classification was proposed: DSM-IV and its revised version, DSM-IV-TR (34, 35). In this version, the diagnostic criteria focused mainly on five different features: Autism, Pervasive Developmental Disorder-Not Otherwise Specified (PDD-NOS), AS, childhood disintegrative disorder (CDD) and Rett syndrome (36).

## From a single autism diagnosis to a broad spectrum

The most salient change from the DSM-IV to a new version DSM-5 (4) has been the understanding of autism as a spectrum varying in severity and functioning. Over time, researchers have used a lot of resources to identify a clear etiology for autism. It seems to us that the most likely “cause” might be a combination of different proteins and genes. Studies found dozens and hundreds of genes related to ASD but not a clear specific single one (36).

Another issue was that the profiles were not consistent. These two reason led to conceptualizing autism as a spectrum (37). The classification is as follows:

- “*Symptoms must be present in the early developmental period”*
- “*Deficits in social-emotional reciprocity* must be present (or have been present earlier in development)”.
- “ASD can be diagnosed *with or without accompanying language impairment”*.
- “Peculiar speech patterns are not required for a diagnosis. However, *echolalia and idiosyncratic phrases* are considered examples of *Stereotyped or repetitive movements, use of objects, or speech*—one of four non-social features”.
- “Any two of the following must be present (currently or earlier in development): (1) *Stereotyped use of objects*; (2) *Insistence on sameness, inflexible adherence to routines, or ritualized patterns of verbal or non-verbal behavior*; (3) *Highly restricted, fixated interests that are abnormal in intensity or focus*; (4) *Hyper- or hypo-reactivity to sensory input or unusual interest in sensory aspects of the environment”*.
- “*Hallucinations and delusions, which are defining features of schizophrenia, are not features of ASD”*.

The eleventh version of the international classification of diseases ICD-11 (2018) is probably the most recent available tool to categorize most conditions that exist among people. ICD-11 is widely used in Scandinavian countries and largely follows the evolution of the DSM-5. Both have merged separate diagnosis into the broader category of ASD, meaning that concepts such as AS have disappeared. However, it should be noted that the ICD-11 still distinguishes between different subtypes of ASD according to the varying levels of cognitive and language functioning. Similarly, in the DSM-5, it is observed that the APA based their classification on the severity of the cases in order to address the needs of each individual and ensure a better daily functioning. This might be an advantage in some cases but might cause a problem in others, especially at the higher end of the spectrum. For the most functional, getting a diagnosis, and thus access to social assistance, might now be harder. In addition, if we take the example of AS, the diagnosis becomes part of their identity. Thus, recategorising these people to the ASD group may be confusing or even insulting as it classes them alongside individuals in the lower end of the spectrum. We should also highlight the fact that ICD-11 considers different subtypes of ASD:

- 6A02.0 Autism spectrum disorder without disorder of intellectual development and with mild or no impairment of functional language.
- 6A02.1 Autism spectrum disorder with disorder of intellectual development and with mild or no impairment of functional language.
- 6A02.2 Autism spectrum disorder without disorder of intellectual development and with impaired functional language.
- 6A02.3 Autism spectrum disorder with disorder of intellectual development and with impaired functional language.
- 6A02.4 Autism spectrum disorder without disorder of intellectual development and with absence of functional language.
- 6A02.5 Autism spectrum disorder with disorder of intellectual development and with absence of functional language.

## Autism spectrum disorder and identity

Being diagnosed with a disorder such as autism cannot help but influence both how one sees oneself and how one is seen by others. In other words, it becomes an *imperative identity*, meaning a facet of one's social self that becomes relevant in almost all situations and over who's content one has only limited control. Identity is often defined as how someone presents themselves, and sees themselves, as belonging to a group (38, 39). We all inhabit multiple identities which may complement or overlap each other, but which may also be in conflict. Not only is the individual's own self-presentation important for identity construction, but also those imposed by the other members of the target group, and society at large. Some identities are more central, and tend to “bleed over” and influence others, and it is these we refer to as imperative identities. This may be problematic, as autism is stigmatized and subject to numerous myths and misunderstandings in the broader culture (40).

The way in which autism is presented in social discourse is of central importance. This is because people cannot by themselves determine the nosological framework within they are classified by society (41). Such representations within public discourse become narratives that define ASD individuals' place in society and how they “should” see themselves (42, 43). Negative opinions of ASD lead some to conceal this identity, where the sharing of this information with others becomes akin to “coming out” (44). Another strategy is to resist the voices of public discourse, either by promoting one's own definition, or by finding a subgroup who also rejects public narratives. In this way, we can see that people with ASD, similar to other developmental disorders, occupy a space where they are exposed to a number of different influences affecting their identity construction. Nevertheless, we are seeing a positive development in that the awareness and knowledge of autism has grown, and it has increasingly become a valid way in which those diagnosed may identify themselves; it has become one of many possible identities. With increased rates of diagnoses, and the gradual fading of stigma, we also see an increased formation of social groups centring on shared experiences of autism. Several studies have discussed the social identity theory and how the individuals within the spectrum hide and camouflage their social and relational difficulties (45–47). The main debate has been whether this camouflaging attempts are stigmatizing or not.

Huynh et al. (48) highlight the nosological social change that results from a new diagnostic criteria for individuals with AS both for them as individuals as well as their social groups.

Since AS was included in the DSM-IV, distinct communities and social milieus have emerged centered around a shared “aspie” identity (49, 50). Such groups, rather than seeing autism as a disorder, present it as a different, but equally valid, part of human diversity. Similarly, the autism rights movement (ARM) argues that behaviors connected to autism should be better understood and respected in society, allowing individuals with ASD to function better in social settings (51). Indeed, part of this advocacy on the part of the ARM was to coin the term aspie as a shared identifying focus. As autism affect many parts of a person's life, such as modes of speech, outlook, interests, etc., it may be argued that they may comprise a separate culture. In this way, a clinical diagnosis has been appropriated by the individuals themselves as a shared and unifying self-identification (49, 52).

As far as we are concerned, and from our personal experience with individuals with ASD, having a diagnosis can either be instructive or destructive. In the first case, a diagnosis can orient the parents, teachers and people working with the child in order to cover more the needs he or she has, adapt to the necessities or something as simple as understanding that person. On the other hand, it is very unfortunate that having this label may risk exposing the child to marginalization or bullying.

## Language and social functioning

Language is such an important tool for social communication and daily social interactions.It is also considered as a tool for us to develop cognition and learn in general. After approaching the identity issue in the previous section, a logical step would be focusing on language abilities.

The most discussed area within ASD is indeed language development. While the diagnostic criteria of both the DSM and ICD stated no “significant evidence” of structural language delay in Asperger Syndrome for instance, scholars who work with AS individuals note difficulties in more complex language domains (53). For instance, once structural language is acquired, these individuals tend to be very literal (54–56). The language of schoolchildren with Asperger syndrome is characterized by following this trend for a pedantic and poorly modulated speech, poor conversation skills, and intense concern on very specific topics. It gives a good development of the formal and structural aspects of language, but fails in the communicative aspects. In certain cases, children can be very quiet, so even parents may have difficulty remembering details of language development. Social relationships and usually start conversations, although they are reduced to their areas of interest using verbiage that the speaker finds it excessive.

Children with ASD not only have a significant delay in language acquisition, but also some evolutionary patterns in this field clearly deviated from the normal process: reduced or no babbling, echolalia, repetitive expressions, limited vocabulary, articulation difficulties and expression. Individuals with ASD have major difficulties in terms of understanding and using intonation patterns to express different content in different communicative contexts. In conversations comments or expressions are often misplaced, omitting the information necessary for the interlocutor to understand.

## The situation of autism in the scandinavian countries

It is no secret that the global number of children diagnosed with autism has increased substantially the last decades (57). Norway, as well as other Scandinavian countries, is no different. Indeed, the Norwegian health registries have reported that 0.9% of children have received an ASD diagnosis by the age of 12 (58). In Sweden, 0.40, 1.74, 2.46, and 1.76% among 0–5, 6–12, 13–17, and 18–27 year olds, respectively, are considered to have an autism diagnosis (59). These authors explain the increase in percentages by the raise of societal awareness and awareness of diagnostic criteria. This suggests that autism has always been among us but has been conceptualized wrongly or mistaken with other disorders since the beginning as discussed earlier. In Denmark, the situation is similar and 1.5% of children have an autism diagnosis by the age of 10 years old (60). The fact that these numbers are substantial highlights both the prevalence of this disorder and the importance of an accurate diagnosis so that the children in question get the help they need.

According to Surén et al. (57), Scandinavian countries are on a good track and are following the individual national regulations, as well as up-to-date diagnostic criteria. The central issue that the authors consider problematic is the lack of assessment for language and adaptive skills despite these being crucial for an autism diagnosis. This is the case because individuals with autism, from the authors' perspective, are characterized by an impaired social interaction and communication. Considering that language is probably the most important “tool” for social interaction and communication, this leads us to question the numbers reported by the different national registers, which in turn illustrates the necessityof extending the diagnostic evaluations to all relevant competences before labeling a person.

### The importance of early intervention: The Norwegian perspective

As a general rule, it is important to improve the brain functions by stimulating the child's mind during the first years due to the great neural plasticity up to 3 years (61). In the case of children with ASD, the early years are crucial for both motor and brain development, in addition to the different language and cognitive skills.

According to Zalaquetta et al. (61) an early intervention may change the child's neural biology. With this it is meant a highly structured and containing environment, as well as a regular and intensive early intervention are likely to “build” new neural networks and connections allowing the child a better adaptation, learning and functioning.

### Educational inclusion in Norway: The autism perspective

In Norway and the other Nordic countries, it is seen as important that all children participate as far as possible in the same activities regardless of their diagnostic profiles. One of the biggest difficulties that we face is related to such “proper” inclusion of children with special needs and their enrolment in the daily social and academic context (62).

Individuals with autism are no different in this regard, and because of their diverse profiles and their impaired social abilities, their inclusion is perhaps even more challenging. Norway has been strongly influenced by the philosophy followed in other European countries. This is clearly stated in the Salamanca agreement (63).

By Norwegian law, school is compulsory for children from the age of 6–16 years old. This applies no matter whether you have a diagnosis or a special need, this is mainly the case because all schools have the obligation to be prepared and ensure proper access to all children aiming always on achieving a complete inclusion and normalization.

### Social and linguistic training as an example followed in Norway for normalizing social functionning

When it comes to early intervention, Norway is no different from most western countries. Early intervention in children with ASD is still a little bit tricky. This is specially the case for girls (who usually display better social and linguistic skills than boys) and individuals in the higher end of the spectrum.

In both Sweden and Norway, there are public programs which provide early intervention for young children with ASD. Both countries use the supervision of special pedagogues and clinical specialists in order to provide an adapted learning environment to the child with ASD (64). So far, the national guidelines for both countries are quite general. There are regulations including all children with disabilities of course but when it comes to the actual intervention, it is a general practice to focus on the individual profile of each child and try to address his needs with the aim of improving at all different levels. As the word “spectrum” suggests, there is a huge variance in profiles dispite a similar diagnosis.

The previous sections discuss the importance of inclusion, particularly its role in creating socially functional individuals. This is a central concept in Norway, as well as the other Scandinavian countries. Early detection is vital in this process, which calls for collaboration between the parents, the kindergartens, the pedagogical-psychological services (PPT) and other relevant instutions in order to address the child's needs. One tool that has been used in Norway is the early and intensive behavioral intervention (EIBI). It has been very effective for two main reasons. On the one hand, it is adapted for children under 5 years of age and on the other hand, it is flexible enough to take the huge variability of the autism spectrum into account, adapting to the specifics of each child. This includes the child's skills as well as his/her needs.

EIBI takes into consideration the present biological and environmental conditions and the potential impact and influence that these can have on the child's functioning, which can include for instance play, social skills, empathy, etc. The main goal is to start simple from simple situations, use these as a basis for developing new skills and abilities and reduce deviant or undesired behaviors (65).

EIBI uses the basic elements of behaviorism to increase positive conducts and lower negative ones such as aggressivity, tantrums, etc. It includes the participation of both parents and kindergarten employees as it can be implemented at home or in the kindergarten. Moreover, EIBI is usually combined with other interventions and therapies depending on each patient.

EIBI's results have been very positive. Different studies report a great evolution in toddlers with ASD where it has been implemented. This suggests that EIBI is one of the most efficient approaches, especially when it comes to teaching children adaptative behaviors (66–68). However, it is not a miracle cure, nor a perfect tool, as Klintwall and Eikeseth (68) claim that dispite EIBI's positive results, several cases could not achieve a “normal” functioning.

The most criticized or what is considered most challenging with the use of EIBI is the fact that the training places great demands on the child and the family. This has created a bit controversy among professionals regarding to which extend can for instance the parents be implicated, and to which extend this concrete issue can be beneficial for the development of the child in question. Is it positive for the child's natural development? (69, 70). Here, different perspectives and critics can be considered: Mottron (71) states that to a certain extent, EIBI intends to eradicate the actual traits that make a determined person that person, and that a behavioral intervention will change the personality of the child. Furthermore, Silberman (69) highlights that a behavioral intervention is rather inflexible, rigid and even intrusive to the child's family unit. Alternatively, Vea et al. (72) acknowledge these but believe that it is all about choosing the appropriate way of method and training implementation. In this line, EIBI should be adopted and integrated into a natural, everyday life context, where the child is not isolated from his/her natural environment, activities and interests.

## Discussion and conclusion

A number of exceptional contributions from several important scientists have helped change the concept of ASD from a mental psychiatric illness to a developmental disorder. Furthermore, cultural, social and political shifts have contributed in the acceptance of these individuals as participants in society and limiting the extent to which they are segregated.

A shift of the understanding of a disorder would be far more visible in natural science perspectives, such as medicine (or chemistry and physics), which are far much easier to measure rather than in cultural fields. This might facilitate the new production of meanings, and this is the case of the ASD conceptualization, since in this article we have tried to highlight how the concept of autism is not static but has been subject of variance depending on time, disciplines, culture, etc.

The most modern conceptualization up to now is the one provided by the DSM-5 and ICD-11. The changes in these classifications are based on scientific evidence rather than political reasons or pressure (73). However, the new classification system rouses some concern, especially with regard to the risk that higly functioning individuals no longer meet the diagnostic criteria for autism.

Several studies seem to confirm these concerns. Vivanti et al. (73) have shown that a significant proportion (10–40 percent) of people who responded to the DSM-IV criteria for diagnosing autism do not respond to the new DSM-5. So, the question becomes which is the right diagnosis? Even if the DSM-5, as well as ICD-11, is based on a more interdisciplinary work and field research, the new diagnosis seems more exclusive rather than inclusive for some individuals.

The fact that most studies focusing on autism spectrum disorder are conducted in western countries might present a big limitation to the current research field. The nosology of autism spectrum disorder has an inmense complexity associated to both its etiology as well as its pathways of development (74). For instance, if we take into consideration that one of the traits we focus on while diagnosing a person within the spectrum is the impaired social interaction, this interaction itself can be biased by cultural differences and traditions that might be different in other cultural context and environments.

Another important issue is the camouflaging strategies that several individuals within the spectrum use to overcome their social interaction difficulties and attempt to avoid to be socially excluded (45–47). This is of vital importance for professionals and clinicians in charge of clinical assessments, who should be aware of this stigma and potentially include camouflaging assessment questions in the evaluation tools (45, 75). Fombonne (46) encourages further research focussed on a clear conceptualization of the spectrum and a clear differentiation between cognitive and psychological aspects (behaviors, processing, social abilities…).

The communication problems can certainly be comorbid with other disorders or diseases. This, coupled with ASD being a spectrum, calls for flexibility and care in order to avoid misdiagnoses.

Even if the DSM-5 and ICD-11 provides a new diagnosis, doubts persist as to their practical utility in terms of treatment strategies and as to whether people diagnosed with this disorder may have access to services tailored to their condition.

Let's take the example of Asperger syndrome. Associations assert that the introduction of Asperger syndrome into a broad, undifferentiated category undermines the identity of the people affected by this syndrome. This concrete idea is supported by the findings in Katz et al. (76) study, who highlight the fact that shifting from autism to autism spectrum disorder creates a certain ambiguity that might create potential malaise in several different settings. However, the vision adopted in the DSM-5, which is to classify ASD depending on the level of support they need, corresponds better to the rights-based vision of the United Nations Convention on the Rights of Persons with Disabilities (UNCRPD). The intrinsic dignity and value of every human being must be fully recognized, whatever the severity of the disability. Therefore, a diagnosis or sub-diagnosis should in no circumstances be the basis for imposing identities on a person or group of people. Similarly, in this definition, the UNCRPD states that no person should be regarded as disabled against their will. In Norway, these rights are facilitated through societal inclusion, for instance the fact that children with ASD usually attend school with their neuro-typical peers.

We consider that Asperger syndrome disappearing into the broader category of ASD also carries a great risk of the people diagnosed losing access to support and services that they need. The risk of this increases with the widespread stereotype that highlty functioning ASD individuals are geniuses with no need of assistance.

The removal of AS from the DSM-5 creates issues for the aspie community. As has been presented, their shared identity was focused around a clinical diagnosis, but which is no longer extant. This raises many questions as to how this change will affect the group's self-identification, and in turn also whether there were ethical considerations that should have been raised when deciding to remove AS as a recognized diagnosis (48).

Another relevant concern is the current male-biased societal understanding of autism spectrum disorder (46). This is a serious issue because it leads to this group not having access to the support and training they are entitled to. An early intervention is always desired in this kind of disorders, and getting a later diagnosis basically means a later intervention. Consequently, this might lead to difficulties during the adolescence period that can extend to adulthood. As an attempt to overcome this male dominance and bias within the spectrum, Lai and Szatmari (26) suggest that increasing awareness and knowledge about this issue might be beneficial, and conversting and translating the experiences and testimonies from girls within the spectrum might be very informative and helpful both to provide a clearer picture when it comes to the diagnosis of girls within the spectrum but also to help optimize the support and social services that the girls within the spectrum may have access to. Moreover, Lai et al. (47) claim that further research involving constructs and measurements, demography, mechanisms, impact and tailored support is necessary in order to get a clearer picture of what the real situation is and how to proceed in the future.

When it comes to EIBI in Norway and the Scandinavian countries in general, relative effectiveness has been proven. However, different issues still must improve. First of all, more grounded research is needed to ensure that the positive results would last to adulthood, second, more research in the methodology is also needed in order to secure a good training of the team involved (educators, parents, and staff in general). This would provide even more optimal results of the treatment (1, 68).

Finally, the increasing percentages of autism diagnosis in Norway and the Scandinavian countries in general should be taken into account. We consider that it is vital to be able to understand the underlying reasons for this, therefore, further research is needed with more specific thresholds. We acknowledge that this might not be very easy considering how broad the spectrum is but we think that we could clearly benefit from a more focussed and replicable research line. The diagnostic labels granted to the different individuals within the spectrum impact the societal views the public (76) and different communities would form on these people. This will of course have great influences on this concrete population's self-esteem, social, psychological and emotional development (41). We also consider that the identity and individual differences should be taken into account, and perhaps knowing the etiology of the disorder might put us a step closer to decipher the autism enigma.

## Author contributions

SC has written the first draft of the manuscript. FS and AP wrote some sections of the article. All authors contributed to the article and approved the submitted version.

## Conflict of interest

The authors declare that the research was conducted in the absence of any commercial or financial relationships that could be construed as a potential conflict of interest.

## Publisher's note

All claims expressed in this article are solely those of the authors and do not necessarily represent those of their affiliated organizations, or those of the publisher, the editors and the reviewers. Any product that may be evaluated in this article, or claim that may be made by its manufacturer, is not guaranteed or endorsed by the publisher.
